# Primacy Effect of Dynamic Multi-Sensory Covid ADV Influences Cognitive and Emotional EEG Responses

**DOI:** 10.3390/brainsci13050785

**Published:** 2023-05-11

**Authors:** Carlotta Acconito, Laura Angioletti, Michela Balconi

**Affiliations:** 1 International Research Center for Cognitive Applied Neuroscience (IrcCAN), Catholic University of the Sacred Heart, Largo Gemelli 1, 20123 Milan, Italymichela.balconi@unicatt.it (M.B.); 2 Research Unit in Affective and Social Neuroscience, Department of Psychology, Catholic University of the Sacred Heart, Largo Gemelli 1, 20123 Milan, Italy

**Keywords:** consumers, dynamic advertising, multisensory perception, EEG, primacy effect

## Abstract

Advertising uses sounds and dynamic images to provide visual, auditory, and tactile experiences, and to make the audience feel like the protagonist. During COVID-19, companies modified their communication by including pandemic references, but without penalizing multisensorial advertising. This study investigated how dynamic and emotional COVID-19-related advertising affects consumer cognitive and emotional responses. Nineteen participants, divided into two groups, watched three COVID-19-related and three non-COVID-19-related advertisements in two different orders (Order 1: COVID-19 and non-COVID-19; Order 2: non-COVID-19 and COVID-19), while electrophysiological data were collected. EEG showed theta activation in frontal and temporo-central areas when comparing Order 2 to Order 1, interpreted as cognitive control over salient emotional stimuli. An increase in alpha activity in parieto-occipital area was found in Order 2 compared to Order 1, suggesting an index of cognitive engagement. Higher beta activity in frontal area was observed for COVID-19 stimuli in Order 1 compared to Order 2, which can be defined as an indicator of high cognitive impact. Order 1 showed a greater beta activation in parieto-occipital area for non-COVID-19 stimuli compared to Order 2, as an index of reaction for painful images. This work suggests that order of exposure, more than advertising content, affects electrophysiological consumer responses, leading to a primacy effect.

## 1. Introduction

In recent years, advertising has shifted from being conceived as a unidirectional communication from companies to consumers to an ongoing, two-way dialogue between the company itself and consumers. This conceptual change has also been characterized by the emergence of multi-sensory advertising, with the aim of providing a ‘360-degree experience’ that engages all the senses and allows the consumer to feel like the protagonist of the scene depicted [1].

Specifically, to provide a multisensory experience, marketers can adopt several strategies, including (i) using dynamic imagery to develop a perception of motion [2], (ii) incorporating a tactile feature [3], (iii) implementing ads based on sounds and music, and (iv) recalling the memory of previously experienced concepts and experiences with an olfactory or taste component [4,5].

Indeed, multisensory cues used in advertising are able to influence consumers’ emotions and cognitions about the advertised product [6]. 

The COVID-19 pandemic has been characterizing the global landscape since 2019 and, after a couple of years, is still impacting various areas of life, both private and public, such as economics, marketing, transportation, business, and human health. The concern about COVID-19’s impact on mental health has also led the World Health Organization (WHO) to seek citizens by providing them with verified information and advice to assist the emotional and psychosocial health of various target groups [7]. The serious effects of the pandemic on several psychological and social elements, such as anxiety and depressive symptomatology, the degradation of living quality, and financial difficulty, have already been raised in numerous research [8,9,10]. In general, uncertainty and fear regarding the threat of contagion were caused by COVID-19 [11].

Even as a result of safety measures, such as social distancing behaviors [12], put in place by people to try to keep the situation under control [13] and limit the spread of the virus [14], the sense of fear has had a significant impact on marketing and consumer behavior [15,16,17,18]. Hoarding, which is defined as buying only the most basic daily goods in large quantities, is certainly one of the consumer-driven trends that had a significant impact on marketing and sales [11,19]. Along with this, there has also been a procrastination attitude toward purchasing and consuming supplementary goods and products that are not considered essential, as well as a preference for making purchases online [19].

Aware of all these new consumer behaviors, advertising must change to remain relevant and effective during these critical periods (i.e., pandemics [20]), but without penalizing the multi-sensory nature. In these years, in fact, different research studied how consumer perceptions of threat influence their opinions of advertising messages [21,22,23]. For example, it was found that during the COVID-19 pandemic there was an increased interest in advertising based on authenticity, defined as something real, genuine, and characterized by credibility and tradition [21]. According to recent studies, authenticity permits to satisfy people’s psychological requirements for safety [22] but also is helpful to reduce perceived risk and ambiguity [23]. In this sense, adopting authenticity in advertising could reduce consumers’ uncertainty and threat, leading to better consumer evaluations [24]. In addition to products perceived as authentic, it has been shown that during the COVID-19 pandemic, consumers preferred familiar and known products that can satisfy the need for security [13], but at the same time, they also went in search of variety because of the need to restore control and freedom [25]. 

Using stimuli with a strong emotional impact is another very popular advertising technique that was also applied during the pandemic, in which many businesses have understood the potential of using the COVID-19 allusion in advertisements as a source of client engagement, to create positive feelings and brand perceptions [26]. This approach is based on the idea that stimuli with a stronger emotional impact are more likely to be remembered [27,28,29]. The importance of using emotional impact in advertising was demonstrated and previous research pointed out that for audiences to understand a message, advertisers must incorporate a certain driving force in the message: this motivation comes from appeal [30]. The purpose of emotional appeal is to persuade customers to receive a favorable reward or avoid punishment [31] and, for this reason, in advertising, it is possible to use positive and negative emotional appeals [32]. 

Although the use of stimuli with a strong positive emotional impact (used to capture customers’ attention and influence purchasing behavior) is generally accepted, there is still room for argument on the efficacy of advertisements with a negative emotional appeal. Indeed, on the one hand, some research has shown how the use of fear, anger, sadness, and guilt in advertisements is useful in inducing changes in behavior [33,34], for example in reducing driving speed [35]. However, the second line of research [36,37] has discovered that using emotional stimuli that are highly emphasized negatively can lead to attitudes of displeasure and avoidance in consumers, as seen, for instance, in Wolburg’s research (2006) on anti-smoking advertisements. 

Another important aspect to consider when evaluating the impact of an advertisement is the order of the stimulus presentation itself. Several psychological studies have extensively examined serial position effects applied to different research domains and, in particular, two order effects have been investigated: the primacy and recency effects [38,39]. The term primacy effect is used to indicate the situation characterized by a greater persuasion because it is the first item presented and has a higher chance of it being recalled than the items in the middle of a list. In contrast, in the recency effect, items at the end of the list are more likely to be recalled and, as a result, are more preferred than other items.

These two-order effects, however, are relatively unexplored in advertising contexts. Only a few researchers, in fact, have attempted to investigate the primacy and recency effects of advertising stimuli [40,41,42,43,44,45,46]. 

In order to study consumer attitudes and affective responses to advertising, a significant contribution is made by consumer neuroscience [47,48,49,50,51,52], combining the use of explicit measures, aimed to collect conscious processes that the participant is able to communicate, with implicit measurements, which can provide insights into unconscious cognitive and emotional processes [52].

One of the many technologies used in the field of neuromarketing to detect implicit measurements is electroencephalography (EEG) [53,54]. Indeed, a lot of neuromarketing research used EEG to investigate consumer behavior in terms of preference and decision-making in different fields, from shoe products [55] to automotive brands [56], or in the luxury fashion industry [57]. 

To determine which brain frequencies and areas of the brain are most involved in consumer preference, Yilmaz and colleagues (2014) found that the low-frequency bands in frontal and temporal areas allow more information [58]. Studies on decision-making further support these findings, indicating that the frontal and centro-parietal regions are most crucial to the decision-making process [53]. The study by Khushaba and colleagues (2012), for instance, demonstrates increased activation of the theta band during the choosing process in the frontal, parietal, and occipital regions, the alpha band in the frontal and parietal regions, and beta in the occipital and temporal regions [59].

This research is motivated by the fact that emotional advertising with a clear reference to COVID-19 has been used since the start of the pandemic and that a prior study examined the effectiveness of such emotional stimuli without examining the effect on behavioral intentions [60]. 

In this context, the current study aims at investigating, with the use of EEG, the effects of dynamic and multi-sensory advertising with information related to COVID-19 on consumer behavior in terms of cognitive and emotional responses toward the brand. Going down to specifics, this work also attempted to investigate the order effect, that is, to see whether or not this effect impacts the type of stimulus observed by the participants.

Firstly, it was expected to find an increase in neural activity for alpha and beta bands during COVID-19 commercials, compared with control commercials, as a marker of increased cognitive engagement when this type of stimulus is presented first. According to some studies, in fact, alpha and beta bands are connected to the maintenance of cognitive engagement [61,62].

Similarly, it was hypothesized to observe a greater emotional involvement represented by an increase in neural activity for the theta band when COVID-19 stimuli are viewed after non-COVID-19 stimuli. Specifically, previous research showed that the theta band is involved in processing stimuli with a high emotional impact [63], such as unpleasant stimuli [64].

## 2. Materials and Methods

### 2.1. Sample

A total of 19 participants, aged 20 to 28 (5 males and 14 females; Mean age = 25.03; Standard Deviation age = 2.04), were recruited in collaboration with the Catholic University of the Sacred Heart, Milan, Italy. 

While the recruitment of a young sample with unreported educational or socioeconomic status could influence the generalizability of the results, it was necessary to identify a sample that was as standardized as possible and consisted of young people who were interested in the field of sports, users than non-users of the investigated brand. Moreover, this sample was chosen because of the possible cultural status of the subjects, which could have influenced the cultural and social issues addressed in the different video stimuli, but also the familiarity with them.

For participation in the study, the following exclusion criteria were considered: (a) psychopathological or neurological disorder, (b) acute medical conditions, (c) head trauma or ongoing psychopharmacological treatment, and (d) post-traumatic stress symptoms connected to COVID-19 experience evaluated with COVID-19-PTSD questionnaire [65]. Participants were right-handed and had normal to corrected vision, and they were mostly graduate and undergraduate students.

The sample was randomly divided into two groups that watched COVID-19-related or non-COVID-19-related advertising in two different orders of presentation: Order 1 (COVID-19 and non-COVID-19) and Order 2 (non-COVID-19 and COVID-19). These two groups, Order 1 (3 males and 7 females; Mean age = 25.60, Standard Deviation age = 2.50) and Order 2 (2 males and 8 females; Mean age = 25.33, Standard Deviation age = 2.23), were comparable for age.

Participants signed a written informed consent and participated voluntarily and without payment in the research. The study was conducted in accordance with the Declaration of Helsinki and was approved by the Ethics Committee of the Department of Psychology, Catholic University of the Sacred Heart, Milan, Italy.

### 2.2. Advertising Stimuli

The stimulus set includes six dynamic and multi-sensory advertisements (three COVID-19-related and three non-COVID-19-related) from Nike, a well-known sportswear company with a strong valence in terms of Corporate Social Responsibility and social advocacy.

The advertisements related to COVID-19 were “Play for the World”, “You Can’t Stop LA” and “You Can’t Stop Us”: in these videos, several allusions to the current difficult pandemic period are mixed with the brand’s typical communications, which are filled with emotive and inspirational elements.

Specifically, the “Play for the World” video represents the first lockdown in 2020, in which people are forced to remain at home due to the restrictions imposed and practice inside their houses: their slow-motion views are alternated with those of deserted playgrounds. The plot “You Can’t Stop LA” compares the societal achievements and failures accumulated in the human match fought against COVID-19 to the wins and losses of the Los Angeles Lakers basketball team. The commercial “You Can’t Stop Us” provides a positive message by emphasizing the storyline of how the world emerged from the lockdown. The split-screen method is employed to produce potent pictures that combine views from various sporting events, as well as images of empty stadiums and individuals discovering alternate ways to practice at home. The importance of returning to live sporting events following the lockdown is emphasized towards the conclusion.

The advertisements related to non-COVID-19 were “What’s your motivation?”, “You can’t be stopped”, and “Steps”: although there are no references to the pandemic, the inspirational and emotional elements that characterize Nike’s communication are still present. 

In particular, the plot “What’s your motivation?” is about a young basketball player who practices a lot and focuses on success, emphasizing that achievement does not come easily or accidentally, but requires preparation. The “You Can’t Be Stopped” video inspires players to find their inner motivations by reminding the audience that when we do, we are unstoppable. The commercial “Steps” narrates the story of a runner’s journey through obstacles and failures.

### 2.3. Procedure

The procedure was characterized by two experimental sessions of about 20 min, identical to each other, except for the visual stimulus shown to participants. In each session, the monitor screen was positioned about 80 cm in front of the participants’ eyes while they were seated comfortably in a darkened room. First, 120 s of EEG resting state baseline were recorded using non-invasive EEG sensors. 

**Figure 1:**
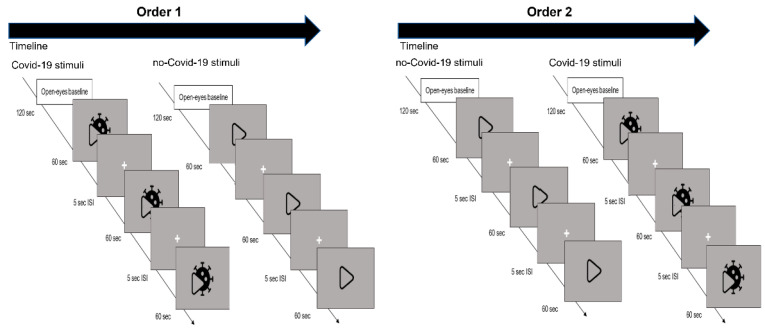
Experimental procedure. The figure exemplifies the chronology of the experiment for each order of stimulus presentation. At the beginning of each experimental session, a 120-s baseline of resting neural activity was recorded with the EEG. In Order 1, three advertisements related to COVID-19 were displayed, followed by three advertisements unrelated to COVID-19. In Order 2, three non-COVID-19-related advertisements were followed by three COVID-19-related advertisements. The stimuli were presented in a randomized order within each order session. The stimuli lasted 60 s and alternated with an interstimulus interval (ISI) of 5 s.

Following the baseline, the participants randomized to Order 1 viewed three videos relating to the COVID-19 condition, whereas the individuals assigned to Order 2 watched advertising unrelated to the COVID-19 condition. In the second experimental session, the participants randomized to Order 1 watched advertisements related to the non-COVID-19, while individuals assigned to Order 2 observed stimuli related to COVID-19. During these experimental sessions, all videos were exhibited randomly and without indication regarding the reference stimulus category (COVID-19 vs. non-COVID-19) in the center of the computer screen, separated by a 5-s inter-stimulus interval during which a black screen was shown (see Figure 1).

Both experimental sessions that define this study were conducted inside the laboratory of Cognitive Psychology at the university where the study was carried out. This experimental setting, specifically, was chosen as a protected environment that was easily controlled in terms of external interferences. In fact, the experiment took place in the sole presence of the experimenter, in complete silence and monitoring any acoustic and light noises.

### 2.4. Electroencephalogram Recording and Data Reduction

EEG activity was collected via an EEG system (LiveAMP, Brain Products, Munich, Germany) with 16 channels placement according to the 10–20 International System [66] over Fp1, Fp2, Fpz, AFF5h, AFF6h, Fz, Cz, C3, C4, T7, T8, Pz, P3, P4, O1, O2. Prior to data collection, electrode impedance for each participant was checked and kept below 5 kΩ. Data were collected using a sample rate of 500 Hz and, following segmentation, were visually examined for ocular, muscular, and movement artefacts. The average power spectra were calculated using the Fast Fourier Transform (Hamming window, resolution = 0.5 Hz) on artifact-free segments. Lastly, average power values were retrieved for the four main EEG frequency bands (Delta = 0.5–3.5 Hz, Theta = 4–7.5 Hz, Alpha = 8–12.5 Hz, and Beta = 13–30 Hz).

For the following statistical analysis, four Regions Of Interest (ROI) were considered: Frontopolar (Fp1; Fz; Fp2), Frontal (AFF5h; AFF6h; Fz), Tempo-central (Cz; C3; C4; T7; T8), and Parieto-occipital (P3; P4; O1; O2).

### 2.5. Statistical Data Analysis

A set of mixed repeated measures ANOVA with Order (2: Order 1 [COVID-19—non-COVID-19], Order 2 [non-COVID—COVID-19]) as the between-subject factor and Condition (2: COVID-19, non-COVID), and ROI (4: Frontopolar, Frontal, Tempo-central and Parieto-occipital) as the within-subject factors were applied on EEG measures. 

For each frequency band (Delta, Theta, Alpha, and Beta) mixed repeated measures ANOVA was performed and post hoc comparisons were applied to the data in case of significant effects. Pairwise comparisons were performed to further examine simple effects for significant interactions, and the Bonferroni correction was applied to reduce the possible bias of multiple comparisons. The degrees of freedom for each ANOVA test were adjusted using the Greenhouse–Geisser epsilon as necessary. Additionally, kurtosis and asymmetry indices were checked to make a preliminary determination of the normality of the data distribution. Computing partial eta squared (*η*^2^) indices have been used to estimate the size of statistically significant effects.

Potential differences related to gender were checked for and excluded. If no statistically significant main and interaction effect including gender were observed, then such variable was not included in the below-reported analyses.

## 3. Results

The next sections show the results for the different frequency bands (see Table S1 in Supplementary Materials for mean and standard deviations).

### 3.1. Theta

**Figure 2:**
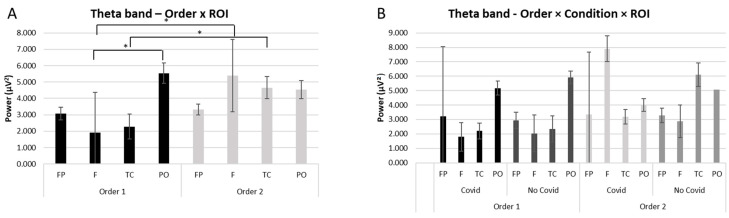
EEG results: Theta. (**A**) Bar graph shows significant differences for theta band activity in region of interest (ROI) between Order 1 and Order 2. Stars mark (*) statistically significant pairwise comparisons. (**B**) Bar graph shows mean trends for theta band activity in regions of interest (ROIs) between Order 1 and Order 2, and compared with the type of stimuli presented. Bars represent ±1 SE.

A significant interaction effect was observed for Order × ROI (F [1,18] = 4.58, *p* ≤ 0.01, *η*^2^ = 0.348). Pairwise comparisons showed higher activity for theta band for Order 2 compared to Order 1 in the frontal ROI (F [1,18] = 4,89, *p* ≤ 0.01, *η*^2^ = 0.377) and in the temporo-central ROI (F [1,18] = 5.43, *p* ≤ 0.01, *η*^2^ = 0.398). Moreover, as shown by pairwise comparisons, an increase of theta power in Order 1 in the PO compared to Frontal ROI (F [1,18] = 4.55, *p* ≤ 0.01, *η*^2^ = 0.354) (see Figure 2A). No other significant differences were observed for the theta band. For descriptive purposes, although not significant, we have reported the bar graph of the interaction Order × Condition × ROI for theta band (see Figure 2B).

### 3.2. Alpha

**Figure 3:**
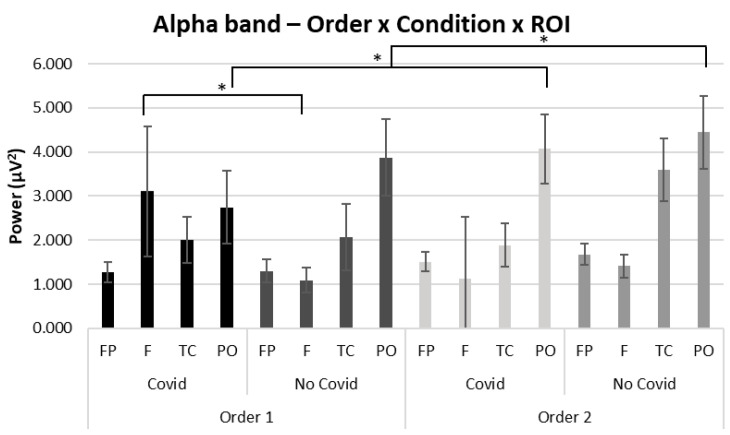
EEG results: Alpha. Bar graph shows significant differences for alpha band activity in regions of interest (ROIs) between Order 1 and Order 2 and compared with the type of stimuli presented. Bars represent ±1 SE. Stars mark (*) statistically significant pairwise comparisons.

For alpha band, a significant interaction effect was observed for Order × Condition × ROI (F [3,34] = 3.55, *p* ≤ 0.05, *η*^2^ = 0.328). Pairwise comparisons revealed an increase of alpha power in the parieto-occipital region in both conditions when stimuli are observed in Order 2 compared to Order 1 (F [1,18] = 4.02, *p* ≤ 0.01, *η*^2^ = 0.370). Moreover, according to pairwise comparisons, greater alpha mean values were found in Order 1 for the frontal ROI in the COVID-19 compared to the non-COVID-19 condition (F [1,18] = 4.32, *p* ≤ 0.01, *η*^2^ = 0.381) (see Figure 3). No other significant effects were found for the alpha band.

### 3.3. Beta

Concerning beta band results, a significant interaction effect was observed for Order × Condition × ROI (F [1,34] = 3.25, *p* ≤ 0.05, *η*^2^ = 0.331). Pairwise comparisons showed an increase of beta power in the frontal ROI for the COVID-19 condition in Order 1 compared to Order 2 (F [1,18] = 4.09, *p* ≤ 0.01, *η*^2^ = 0.350). Further, according to pairwise comparisons, greater beta mean values were found in the PO ROI in the non-COVID-19 condition in Order 1 compared to Order 2 (F [1,18] = 3.89, *p* ≤ 0.05, *η*^2^ = 0.341).

**Figure 4:**
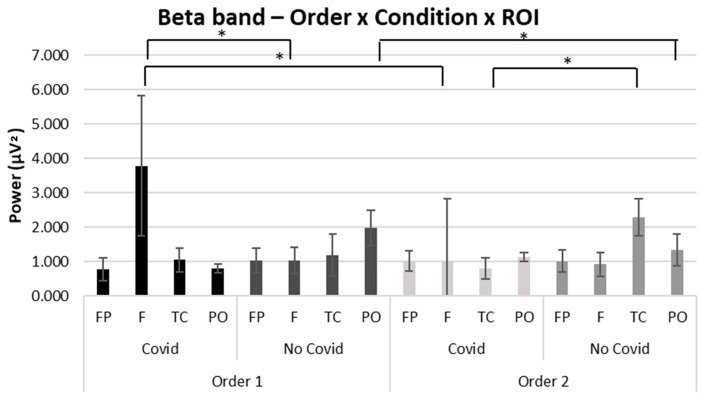
EEG results: Beta. Bar graph shows significant differences in beta band activity in regions of interest (ROIs) between Order 1 and Order 2 and compared with the type of stimuli presented. Bars represent ±1 SE. Stars mark (*) statistically significant pairwise comparisons.

Moreover, pairwise comparisons revealed greater beta mean values were found in Order 1 in the frontal ROI for the COVID-19 compared to the non-COVID-19 condition (F [1,18] = 3.77, *p* ≤ 0.05, *η*^2^ = 0.347). On the other hand, greater beta mean values were found in Order 2 in the temporo-central ROI for the non-COVID-19 compared to the COVID-19 condition (F [1,18] = 4.09, *p* ≤ 0.01, *η*^2^ = 0.391) (see Figure 4). No other significant effects were found for the beta band.

### 3.4. Delta

For the Delta band, no significant effects were found, as the performed statistical analyses reported a significance greater than *p* > 0.5.

**Figure 5:**
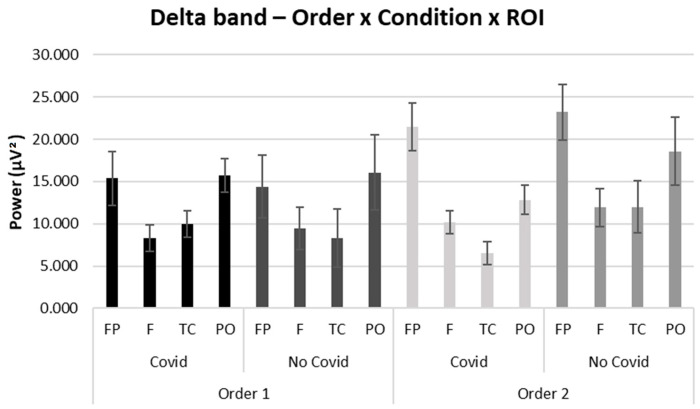
EEG results: Delta. Bar graph shows mean trends for delta band activity in regions of interest (ROIs) between Order 1 and Order 2 and compared with the type of stimuli presented. Bars represent ±1 SE.

For descriptive purposes, although not significant, we have reported the bar graph of the interaction Order × Condition × ROI for delta band (see Figure 5).

## 4. Discussion

The current study explored the cognitive and emotional responses of participants following exposure to dynamic and multi-sensory advertising stimuli characterized by references to the COVID-19 pandemic. In this work, the main significant results were observed for the theta, alpha, and beta EEG frequency bands. Below, these results will be discussed starting from the comparison between the two orders in which the stimuli were presented to the participants.

First of all, at the cortical level, an increase of the theta band was mainly found in frontal and temporo-central areas, comparing Order 2 to Order 1, regardless of the stimuli presented. Several studies found that the theta band is generally connected to the discrimination of affective valence in visual cues [63,67,68,69,70,71,72] and in emotion regulation systems [73]. Specifically, the manifestation of theta band over frontal brain areas has previously been demonstrated to be connected to cognitive control over salient emotional stimuli [67], such as novel stimuli, conflicts, and errors [74], but also painful unpleasant stimuli [64]. Similarly, the temporo-central brain regions are also involved in processing stimuli with high emotional impact [63]. According to the functional meaning of the theta band, it might be plausible that Order 2 (i.e., viewing non-COVID-19 stimuli followed by COVID-19 stimuli) elicits a greater emotional impact requiring cognitive control.

Additionally, a significant presence of theta activity in parieto-occipital compared to frontal regions was observed in Order 1, that is when COVID-19 stimuli were observed before non-COVID-19 stimuli. According to Lang and colleagues (1998), parieto-occipital areas are identified in response to the visual aspects of the stimuli or the arousing levels of the emotional visual stimuli [75]. This result might suggest the relevance of posterior regions in processing the order of exposure: seeing COVID-19 stimuli before non-COVID-19 stimuli would seem to activate an emotional brain response in relation to visual stimuli that are probably perceived as high impact.

A second significant finding related to the alpha band was observed, for which an increase of alpha power was mainly found in the parieto-occipital area in Order 2 compared to Order 1 regardless of stimulus conditions. According to Fu and colleagues (2021), alpha power in the parieto-occipital area could be analyzed as an anticipatory mechanism of visual attention [76]. This result could suggest that Order 1 might be responsible for increased cognitive engagement.

Moreover, with reference to the alpha band, in Order 1 (COVID-19—non-COVID-19) was observed a significant increase of alpha power in the frontal area when COVID-19 compared to non-COVID-19 stimuli are viewed. These findings are consistent with earlier studies suggesting the presence of an alpha frequency band when regions response (in this case, the frontal area) is deactivated [77]. Therefore, it might be possible that during the vision of COVID-19 stimuli there could be a decrease in attention, as suggested by the presence of an alpha band in frontal regions, that might be associated with a lesser positive valence attributed to COVID-19 compared to non-COVID-19 stimuli.

Finally, comparing the two orders in terms of beta band revealed orders revealed two significant results. First, the frontal area of the brain was more activated for COVID-19 stimuli in Order 1 (COVID-19— non-COVID-19) than in Order 2. According to prior research, frontal neuronal activity in the beta band has been connected to the maintenance of the cognitive state [61], and from this point of view is possible to underline how the first stimuli have a greater cognitive impact.

Additionally, Order 1 (COVID-19— non-COVID-19) showed greater cortical activation for the non-COVID-19 stimuli than Order 2 in the parieto-occipital area. This outcome supports the findings that beta reactions are substantially higher for painful images than for happy and neutral images [78].

A significant increase of the beta band also occurred within Order 1 in the frontal area during the viewing of COVID-19 stimuli. Viewing COVID-19 stimuli initially and then non-COVID-19 stimuli can be interpreted as a measure of both sustained attention and the maintenance of cognitive engagement [61,62], as well as of the inhibitory control process [62,79,80]. In Order 2, however, results showed increased activation of beta in the temporo-central areas following the viewing of non-COVID-19 stimuli. Considering that the non-COVID-19 stimuli are the first to be observed in Order 2, this result can be interpreted as showing increased activation of central temporal areas, which may signal the beginning of sensory processing [81], as well as a response to environmental and social stimuli [82].

Considering the results obtained, it is also important to emphasize the possible practical application of this research.

The data show how the use of dynamic and multi-sensory stimuli are able to impact the cognitive and emotional processes of the audience, and, therefore, it could be useful to use this type of communication in the educational sphere to increase awareness and involvement in certain topics, also considering the enhancement for advertising with a social purpose.

In addition, another possible application might involve using this type of stimulus not only for commercial purposes, but also with a view to communicating messages in health and wellness to increase, for example, patient engagement and disseminate correct information about prevention and treatment to every level of the population. Indeed, in this sense, the pandemic has proven to be an optimal context for testing these potential effects.

Despite the work’s innovativeness, some limitations could be addressed. First, explicit emotional judgments were not taken into account in this work; rather, emotional responses were only assessed by exploiting the functional significance of neurophysiological markers. To further understand how explicit and implicit attitudes interact during the consumer decision-making process, future studies should include an explicit assessment of these dimensions by employing, for instance, self-report measures. Second, no behavioral data were gathered in the work to evaluate explicit judgments developed after the experimental manipulation or behavioral attitude changes. Prospective research could further explore whether such a commercial strategy is also capable of successfully altering consumer attitudes toward the brand and, ultimately, boosting their propensity to purchase goods from the advertised brand. Third, to better understand implicit processes, it could be appropriate to integrate this evidence with information collected through multiple neurophysiological techniques, such as EEG integrated with functional Near Infrared Spectroscopy (fNIRS) and autonomic measures recording. Similarly, it might also be interesting to investigate the cognitive processing of visual stimuli using the eye tracker. In fact, the analysis of fixations and saccades related to eye movements during the viewing of a stimulus makes it possible to investigate how advertising affects visual attention and cognitive load.

In consideration of the identified stimuli and from a research perspective, it is important to note that different brands and companies may have specific effects that can influence experimental results. For this reason, future research might consider including advertisements from other companies to fully evaluate the effect of advertising on emotion and cognition. Despite this possible limitation, however, it is also important to emphasize that brand-specific effects, such as cognitive perceptions, are to be considered marginal variables with respect to the intrinsic characteristics of the stimuli used in validation. Indeed, the selected advertising materials were evaluated and validated for the following perceptual characteristics: duration, fps, size, brightness, and content.

Finally, it should be noted that the sample size considered for this study was relatively small and, therefore, in future studies the number of participants should be increased to improve the representativeness and reliability of these findings. Similarly, to increase the generalizability of the results, it might be appropriate to recruit samples from different and multiple sources, and that differ from the category of the university students. Lastly, given the gender unbalances in the sample groups of this study, future research should consider this variable and propose more homogeneous groups.

## 5. Conclusions

To summarize, the present study permitted studying how exposure to COVID-19 dynamic and multi-sensory advertising influences neurophysiological markers of consumers’ cognitive and emotional responses. This work highlighted the significance of the order in which stimuli are shown when participants are exposed to advertising. These findings showed that when comparing orders, the stimulus type —COVID-19 or non-COVID-19— is not so relevant as the order of exposure (i.e., as the one that was observed first), in this way leading to a sort of primacy effect. Another important finding concerns the presence of neurophysiological markers of emotional processing in Order 1 (i.e., COVID-19 stimuli followed by non-COVID-19 stimuli) represented by the significant presence of theta band, while in Order 2 (i.e., non-COVID-19 stimuli followed by COVID-19 stimuli), a cognitive response going on was observed as suggested by the alternate presence of alpha and beta bands. Finally, this study supports previous findings on the importance of employing dynamic stimuli that can activate a multi-sensory experience in the audience in order to impact the consumer’s cognitive and emotional components [1,6].

## Data Availability

The data presented in this study are available on request from the corresponding author.

## Supplementary Materials

The following supporting information can be downloaded at: https://www.mdpi.com/article/10.3390/brainsci13050785/s1. Table S1: Descriptive data related to the significant electroencephalographic results for alpha, beta, theta, and delta frequency bands (Mean ± Standard Deviation).
