# Supplementary material for: Primacy Effect of Dynamic Multi-Sensory Covid ADV Influences Cognitive and Emotional EEG Responses
Source: Brain Sci. 2023 May 11;13(5):785. doi: 10.3390/brainsci13050785 (PMC10216398; doi:10.3390/brainsci13050785)
Supplement: Supplementary file 1 [file brainsci-13-00785-s001.zip › brainsci-2371414-supplementary.pdf]

Table S1. Descriptive data related to the significant electroencephalographic results for alpha, beta, theta, and delta frequency bands (Mean ± Standard Deviation)

Frequency bands	Order	ROI	Covid-19	Non-Covid-19
Alpha band	*Order 1*	Frontopolar	1.27 ± 0.23	1.30 ± 0.26
		Frontal	3.11 ± 1.47	1.09 ± 2.82
		Tempo-central	2.00 ± 0.52	2.07 ± 0.76
		Parieto-occipital	2.75 ± 0.82	3.87 ± 0.87
	Order 2	Frontopolar	1.51 ± 0.22	1.68 ± 0.25
		Frontal	1.13 ± 1.40	1.41 ± 0.27
		Tempo-central	1.88 ± 0.49	3.60 ± 0.72
		Parieto-occipital	4.07 ± 0.78	4.45 ± 0.82
Beta band	*Order 1*	Frontopolar	0.78 ± 0.34	1.04 ± 0.36
		Frontal	3.78 ± 2.04	1.04 ± 0.39
		Tempo-central	1.05 ± 0.35	1.18 ± 0.61
		Parieto-occipital	0.80 ± 0.14	1.98 ± 0.52
	Order 2	Frontopolar	1.01 ± 0.30	1.01 ± 0.32
		Frontal	0.99 ± 1.83	0.92 ± 0.34
		Tempo-central	0.80 ± 0.31	2.28 ± 0.54
		Parieto-occipital	1.13 ± 0.12	1.34 ± 0.46
Theta band	*Order 1*	Frontopolar	3.21 ± 0.49	2.94 ± 0.49
		Frontal	1.79 ± 4.84	2.04 ± 0.55
		Tempo-central	2.21 ± 0.99	2.35 ± 1.27
		Parieto-occipital	5.17 ± 0.55	5.92 ± 0.91
	Order 2	Frontopolar	3.36 ± 0.44	3.29 ± 0.44
		Frontal	7.91 ± 4.33	2.88 ± 0.49
		Tempo-central	3.19 ± 0.89	6.12 ± 1.14
		Parieto-occipital	3.99 ± 0.49	5.07 ± 0.82
Delta band	*Order 1*	Frontopolar	15.40 ± 3.19	14.39 ± 3.70
		Frontal	8.33 ± 1.55	9.48 ± 2.52
		Tempo-central	9.97 ± 1.54	8.29 ± 3.43
		Parieto-occipital	15.73 ± 1.95	16.05 ± 4.45
	Order 2	Frontopolar	21.50 ± 2.85	23.23 ± 3.31
		Frontal	10.20 ± 1.38	11.96 ± 2.25
		Tempo-central	6.51 ± 1.38	12.00 ± 3.07
		Parieto-occipital	12.82 ± 1.74	18.60 ± 3.98
